# Post-transcriptional regulation of photosynthetic genes is a key driver of C_4_ leaf ontogeny

**DOI:** 10.1093/jxb/erw386

**Published:** 2016-10-18

**Authors:** Nicklaus Fankhauser, Sylvain Aubry

**Affiliations:** 1 Clinical Trials Unit, University of Bern, Finkenhubelweg, Bern, Switzerland; 2 Institute of Plant and Microbial Biology, University of Zürich, Zollikerstrasse, Zürich,Switzerland

**Keywords:** C_4_, Cleomaceae, EISA, leaf ontogeny, photosynthesis, post-transcription

## Abstract

C_4_ photosynthesis allows highly efficient carbon fixation that originates from tightly regulated anatomical and biochemical modifications of leaf architecture. Recent studies showed that leaf transcriptome modifications during leaf ontogeny of closely related C_3_ (*Tarenaya hassleriana*) and C_4_ (*Gynandropsis gynandra*) species within the Cleomaceae family existed but they did not identify any dedicated transcriptional networks or factors specifically driving C_4_ leaf ontogeny. RNAseq analysis provides a steady-state quantification of whole-cell mRNAs but does not allow any discrimination between transcriptional and post-transcriptional processes that may occur simultaneously during leaf ontogeny. Here we use exon–intron split analysis (EISA) to determine the extent to which transcriptional and post-transcriptional processes are involved in the regulation of gene expression between young and expanded leaves in both species. C_4_-specific changes in post-transcriptional regulation were observed for genes involved in the Calvin–Benson cycle and some photosystem components but not for C_4_ core-cycle genes. Overall, this study provides an unbiased genome-wide insight into the post-transcriptional mechanisms that regulate gene expression through the control of mRNA levels and could be central to the onset of C_4_ photosynthesis. This mechanism is cytosolic which implies cell-specific modifications of mRNA stability. Understanding this mechanism may be crucial when aiming to transform C_3_ crops into C_4_ crops.

## Teaser

An analysis of exonic and intronic signatures revealed that there was post-transcriptional regulation of cytosolic transcripts during C_4_ leaf ontogeny.

## Introduction

C_4_ photosynthesis is one of the most efficient carbon-fixing reactions on Earth (Hatch and Slack, 1966). It is widespread in many ecosystems and has existed since the late Miocene (Ehleringer *et al.*, 1997). Based on ancestral C_3_ photosynthesis, it is a fantastic example of convergent evolution in response to variable atmospheric CO_2_ in angiosperms (Aubry *et al.*, 2011; Williams *et al.*, 2012). Interestingly, the independent evolution of at least 18 C_4_ plant families seem to be constrained to a recurrent sequence of small evolutionary steps that progressively increased the carbon concentration in the vicinity of the leaf vasculature (Heckmann *et al.*, 2013; Williams *et al.*, 2013). Briefly, in order to favour the ribulose 1,5-*bis*phosphatase carboxylase/oxygenase (RuBisCO) carboxylation reaction over its undesired oxygenation reaction, C_4_ plants concentrate CO_2_ around the enzyme and thereby drastically limit photorespiration (and therefore increase photosynthetic activity). In order to allow carbon concentration, the carbon cycle is segregated into two compartments, each containing one of the two carboxylases involved: the phospho*enol*pyruvate carboxylase (PPC) in mesophyll (M) cells and RuBisCO in bundle-sheath (BS) cells. After diffusing into the leaf through stomata that have acquired the ability specifically to regulate their opening in otherwise unfavourable CO_2_ and humidity conditions (Aubry *et al.*, 2016), CO_2_ is primarily fixed by the O_2-_insensitive PPC. It is subsequently exported as a four carbon compound to the BS cells where a decarboxylase charges the Calvin–Benson cycle (CBC) to feed the final carboxylation by RuBisCO. The biochemistry of various subtypes of C_4_ photosynthesis is classified by one of the three different decarboxylases recruited (Furbank, 2011).

Several complementary studies using high-throughput transcriptomics approaches performed multiple comparisons of steady-state transcript levels between C_3_ and C_4_ leaves as well as during their ontogeny (Brautigam *et al.*, 2010; Gowik *et al.*, 2011; Aubry *et al.*, 2014*a*; Külahoglu *et al.*, 2014). These ‘omics’ approaches are aimed at identifying new elements of transcriptional networks that regulate the C_4_ pathway (reviewed in Burgess and Hibberd, 2015). However, they also confirmed the assumption that multiple levels of regulation—transcriptional (Brown *et al.*, 2011), post-transcriptional (Patel and Berry, 2008), and post-translational (Pick *et al.*, 2011)—contribute to the onset and maintenance of the C_4_ metabolic system (Hibberd and Covshoff, 2010).

Modifications in gene expression are thought to be associated with many of the C_4_-specific anatomical or biochemical features, such as increased vein density, changes in stomata regulation, chloroplast dimorphism, chloroplast positioning, BS suberization, and endoreduplication (Li *et al.*, 2010; Majeran *et al.*, 2010; Gowik *et al.*, 2011; Pick *et al.*, 2011; Aubry *et al.*, 2014*a*, 2016; Külahoglu *et al.*, 2014). Further studies focused on differences in the transcriptome between specialized leaf cell types—bundle-sheath (BS) and mesophyll (M) cells—and seem to confirm the function of transcriptional regulation for gene expression tuning in C_4_ cycle regulation but, so far, without identifying any dedicated transcriptional networks (Li *et al.*, 2010; Chang *et al.*, 2012; John *et al.*, 2014; Aubry *et al.*, 2014*a*; Burgess and Hibberd, 2015). It is noteworthy that, in C_4_ leaves, cell-specificity is not limited to the core C_4_ cycle (i.e. from the initial ${\text{HCO}}_{3}^{–}$ fixation until the RuBisCO carboxylation step) but applies to other parts of the photosynthetic apparatus. In *Flaveria* and *Cleome*, transcriptional signatures show a clear segregation of photosystem I (PSI) and photosystem II (PSII) elements between BS and M cells (Gowik *et al.*, 2011; Aubry *et al.*, 2014*a*). In C_4_ species, from the NADP malic enzyme (NADPME)-subtype (like *Flaveria* and maize) BS chloroplasts are mostly agranal and there is a strong transcriptional investment in ATP production by cyclic electron flow that includes genes encoding proteins of PSI, cytochrome *b*_6_/*f* and NDH (NADH dehydrogenase) complexes (Nakamura *et al.*, 2013; Aubry *et al.*, 2014*a*). RuBisCO transcript levels (and most other CBC transcripts) are largely reduced in C_4_ compared with C_3_ leaves (Ku *et al.*, 1979; Bräutigam *et al.*, 2010). This reduction in RuBisCO is thought to be one of the major advantages of C_4_ nitrogen use efficiency (Oaks, 1994; Ghanoum *et al.*, 2005). Mechanisms that restrain RuBisCO from accumulating in BS are complex and involve a mixture of transcriptional and post-transcriptional features (Berry *et al.*, 2016). In both monocots and dicots, BS-specific expression of RuBisCO is under robust post-transcriptional control. Thus, during leaf development, expression of *RbcS* and *RbcL* have been shown to be uncoupled from translation (Patel and Berry, 2008) and *RbcS* untranslated regions (UTR) are suggested to play a role in RNA stability during the later stages of leaf development. Post-transcriptional regulation of gene expression may not be restricted to RuBisCO but may also be a more widely used strategy for other (photosynthesis-related) gene regulation, especially during C_4_ leaf development.

In order to assess the involvement of post-transcriptional regulation of gene expression, we took advantage of comparative experiments on closely related species during leaf ontogeny in both C_3_ and C_4_ leaves (Külahoglu *et al.*, 2014) using a computational approach called exon–intron split analysis (EISA). EISA takes advantage of the presence of both mature and intron-containing pre-mRNAs in RNAseq samples to discriminate transcriptional from post-transcriptional effects on gene expression (Gaidatzis *et al.*, 2015). While the abundance of transcripts may vary between two experimental conditions (such as here during leaf development), a gene that is exclusively subjected to transcriptional regulation should show tightly correlated proportions of reads mapping to introns and exons. By contrast, a gene that is subjected to post-transcriptional regulation is expected to exhibit a decreased amount of reads mapping to exons compared with reads mapping to introns, as only mature intron-free cytosolic mRNA should be impacted. This has been shown to be a possible proxy to predict gene regulation status (Gaidatzis *et al.*, 2015). For example, a significant increase in intronic RNA expression without an increase of corresponding exonic RNA has been observed in some animal systems after antioxidant treatment, even if the physiological significance of such mechanisms remains unclear (Menon *et al.*, 2016). We applied EISA to leaf development transcriptome series from *Tarenaya hassleriana* (C_3_) and *Gynandropsis gynandra* (C_4_), two closely related species within the Cleomaceae (Külahoglu *et al.*, 2014). Despite some anatomical and biochemical modifications, gene expression profiles of the two species appeared relatively well conserved during leaf ontogeny (Aubry *et al.*, 2014*a*; Külahoglu *et al.*, 2014). Nevertheless genes encoding photosynthetic components and CBC enzymes appear to be subject to post-transcriptional regulation at the level of mRNA accumulation. This process is likely to be a key driver of C_4_ leaf ontogeny.

## Materials and methods

### Data access

RNAseq data were obtained from NCBI (accessions: SRP036637 and SRP036837, for *G. gynandra* and *T. hassleriana*, respectively).

The reference genome for Arabidopsis was accessed from Phytozome (http://www.phytozome.net) with the most up-to-date gene model. The genome sequences from *G. gynandra* and *T. hassleriana* were accessed as described previously (Aubry *et al.*, 2016; Cheng *et al.*, 2013).

### Processing of the reads and quantification of exonic and intronic expression levels

The EISA method was implemented to obtain expression values for reads mapping to introns or exons as published (Gaidatzis *et al.*, 2015) with the following criteria used. Only transcripts that map to a unique position in the genome were considered. Only reads that map inside the body of the gene (no UTRs) were taken into account and only introns enclosed by exons were considered. Exon/intron counts were quantified using qCount from QuasR (Lun *et al.*, 2016). A threshold requiring at least two reads for every exon and intron was applied. Normalization based on the total number of reads for each library was performed separately for exons and introns. Overlapping genes were not considered. ΔExon and ΔIntron were defined as the difference in log_2_ of exonic or intronic expression levels between respective compartments. The model for statistical significance of differential post-transcriptional regulation was implemented in edgeR (Lun *et al.*, 2016). Generally, the lack of reliable genome annotation, especially a definition of exon–intron boundaries, can limit the interpretation of our data (Weber, 2015). We validated our approach using publically available transcriptome data from the *ago1-27* mutant (accession number: GSE77211, see Supplementary Table S1 at *JXB* online). This analysis showed that suppression of ARGONAUTE1 (AGO1) protein, a core component of the gene-silencing machinery (Morel *et al.*, 2002; Vaucheret *et al.*, 2004) modifies the extent of post-transcriptional regulation of 216 genes, especially RNA-metabolic genes (GO term enriched: RNA splicing, RNA processing, and RNA metabolism, Supplementary Table S1). It should be noted that the number of biological replicates and the coverage of the sequencing are crucial when intending to use EISA for a comprehensive evaluation of post-transcriptional regulation of gene expression.

### Analysis of differential gene expression and gene ontology enrichment

Reads from RNAseq libraries of young and expanded leaves were aligned to protein-coding transcripts from the respective genomes of *G. gynandra* and *T. hassleriana* (Cheng *et al.*, 2013; Aubry *et al.*, 2016). Transcript expression was quantified using RSEM version 1.2.30 (Li and Dewey, 2011). Posterior probability of differential expression (PPDE) between the two conditions was estimated using the empirical Bayesian approach implemented in EBSeq version 1.1.6 (Leng *et al.*, 2013). Gene ontology enrichment was performed using a corrected Benjamini–Hochberg enrichment score implemented in Pageman (Usadel *et al.*, 2006).

## Results and discussion

### Application of exon–intron split analysis to plant datasets

**Fig. 1.**
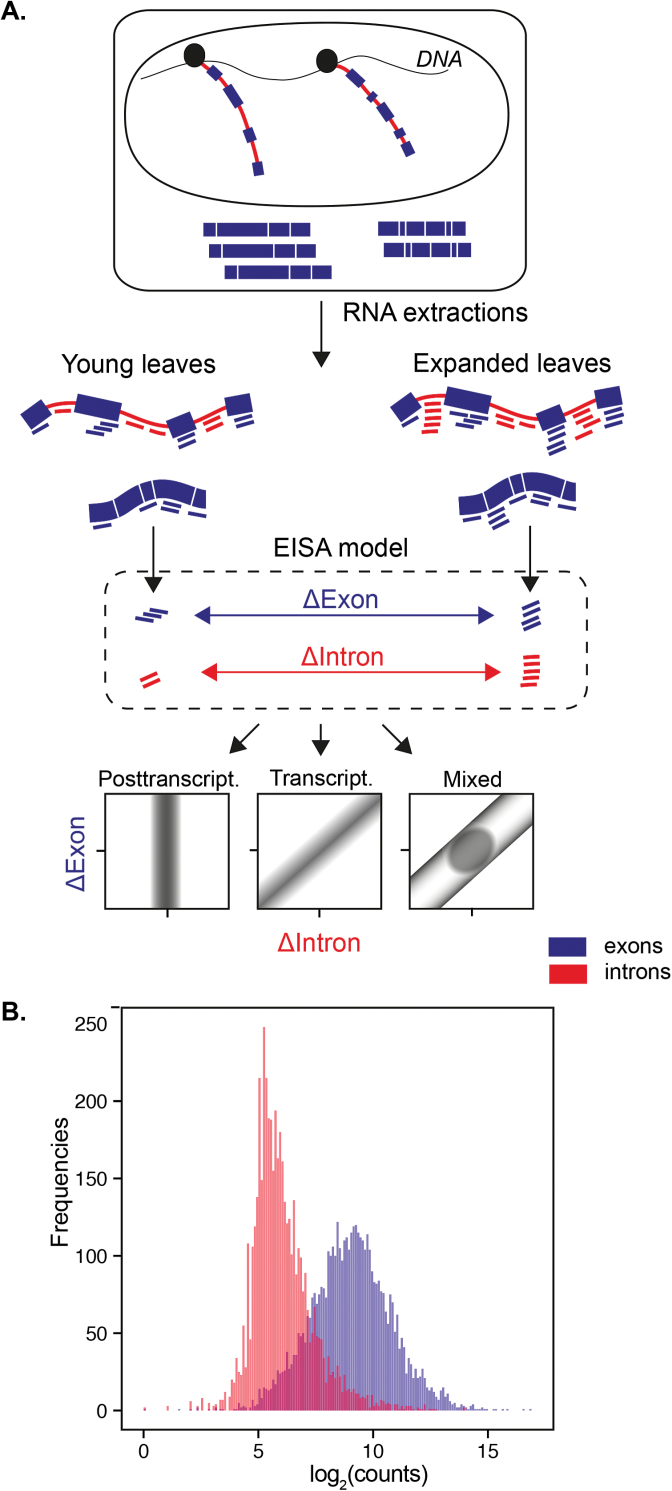
The exon–intron split analysis (EISA) detects the extent of gene expression regulation under post-transcriptional control between two experimental conditions. EISA was applied during leaf ontogeny between young and expanded leaves of *G. gynandra* and *T. hassleriana*. (A) Pre-mRNA comprising introns (red) and exons (blue) are transcribed in the nucleus, then spliced and translocated to the cytosol where the mature mRNA will be translated. EISA counts the differences in intronic (ΔIntron) and exonic (ΔExons) levels between the experimental conditions and compares them as a measure of post-transcriptional controls. The bottom part shows plots expressing ΔExon as a function of ΔIntron to show the possible patterns that are representative of the predominant mechanisms regulating gene expression (mostly transcriptional, post-transcriptional or a mixture of the two). The schematic representation was adapted from Gaidatzis *et al.* (2015). (B) RNA extraction results in a majority of reads mapping to exons but also reads originating from intronic segments.

During standard RNAseq library preparation, the total extraction of mRNAs results in a mixture of mature cytosolic RNA and nuclear nascent or unspliced RNAs (Fig. 1A). Even if the vast majority of the reads analysed originate from exons, a fraction of the reads maps as introns (Fig. 1B). These might originate from genomic contamination, wrong gene models (mis-annotated exons) or from premature RNAs. Levels of intron-specific reads have been proposed to correlate with transcriptional activity and to be used as a proxy for analysing transcription (Zeisel *et al.*, 2011; Hendriks *et al.*, 2014). Intronic read levels often, but not always, correlate with exonic reads when compared across experimental conditions. In the case of post-transcriptionally regulated genes that are for example under miRNA control, the relative level of mature transcripts declines as the silencing machinery exclusively acts on cytosolic mature RNAs. It is of note that there are fewer intronic reads in the preparation when mRNA is selected by poly-A purification compared with total RNA extractions by ribosomal RNA depletion (Trapnell *et al.*, 2010). However, data that are derived from both methods are still suitable for EISA, given a sufficient coverage of the transcriptome (Gaidatzis *et al.*, 2015).

The EISA method, by quantifying reads mapping exclusively to either introns or exons (see Materials and methods for details on filtering), aims to identify transcripts for which the stoichiometry between intron and exon is lost when comparing two experimental conditions (Fig. 1A). In the present study, it was possible successfully to apply EISA to recent congeneric Cleomaceae whole leaf gradient data (Külahoglu *et al.*, 2014) but cell-specific data sets were not sequenced deep-enough to provide a statistically robust result (data not shown; Li *et al.*, 2010; Chang *et al.*, 2012; Aubry *et al.*, 2014*a*; John *et al.*, 2014).

### Genome-scale prediction of transcriptional and post-transcriptional regulation in Cleomaceae leaf gradients

Leaf ontogeny variations between C_3_ and C_4_ leaves observed from comparisons between congeneric species have been well documented for *Cleome*, *Flaveria*, and *Alloteropsis* species (Gowik *et al.*, 2011; Christin *et al.*, 2012; Külahoglu *et al.*, 2014). Here we used publically available transcriptomic data from congeneric Cleomaceae leaf gradients (Külahoglu *et al.*, 2014). For each species, we selected the extreme stages of the leaf gradient (stage 0: ‘Young’ and stage 5: ‘Expanded’) analysed in the study (Külahoglu *et al.*, 2014). 8 855 (37% of all genes detected) and 6 672 genes (28%) were differentially expressed between these two conditions in *G. gynandra* and *T. hassleriana*, respectively. EBSeq was applied using a posterior probability of being differentially expressed ≥0.95 and a minimal fold change of 2 between the two conditions. The results are summarized in Table 1 and Supplementary Table S2).

**Table 1.** *Genes that were detected and that have been filtered (minimal coverage of two reads per exon or intron) by the EISA pipeline and the number of genes differentially transcribed between young and expanded leaves (FDR ≤0.05) in the two Cleomaceae species* The proportion of genes DT compared with the total number of genes can be found within the brackets. Differential expression of genes was calculated between young and expanded leaves using EBSeq. Overlaps between EISA and EBSeq output are shown in Fig. 2.

Differential transcription (EISA)	*G. gynandra*	*T. hassleriana*
Total no. of genes	23 340	22 227
Genes selected for EISA	4 769	5 720
Post-transcriptionally regulated (FDR ≤0.05)	678 (15%)	263 (5%)
Differential expression (EBSeq)		
No of genes DE (FC ≥2, PPDE ≥0.95)	8 855	6 672
Up in young leaves	3 949	2,756
Up in expanded leaves	4 906	3,916

**Fig. 2.**
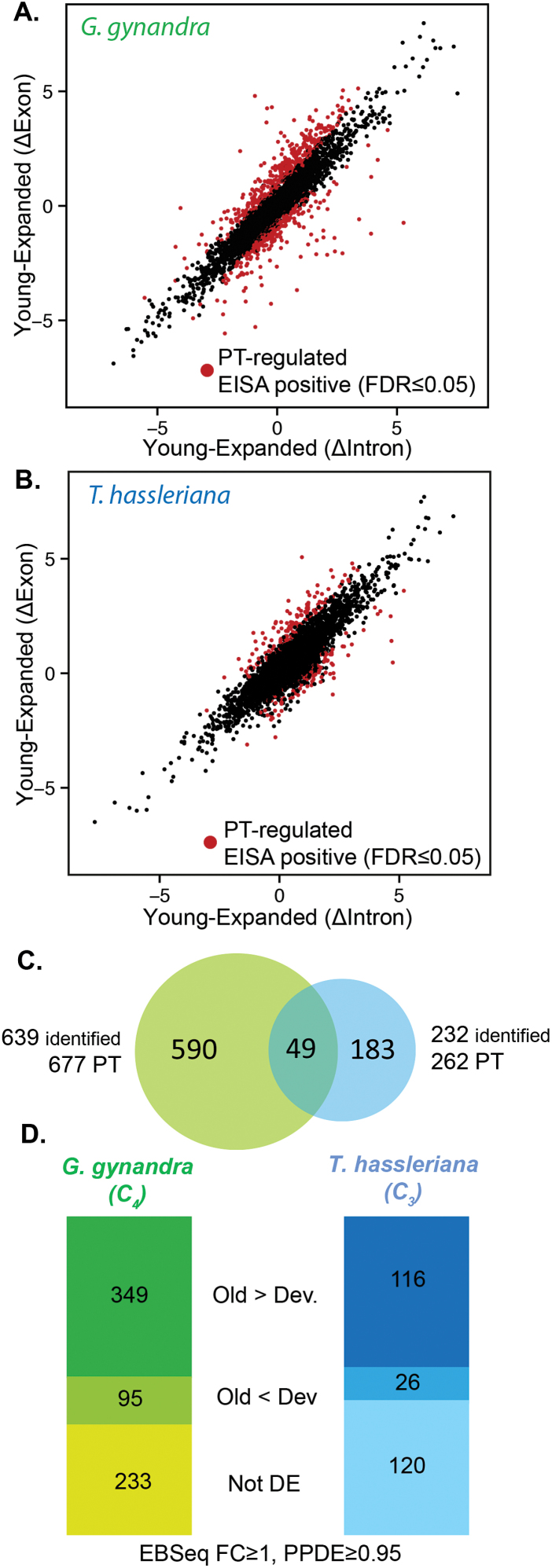
Changes in intronic and exonic levels during leaf development for (A) *G. gynandra and* (B) *T. hassleriana*. Significantly up- or down-regulated genes after EISA analysis (FDR ≤0.05) are shown in red. (C) Overlap between the genes that were post-transcriptionally regulated and identified as potential orthologues by BLAST analysis across the two species. (D) Among post-transcriptionally regulated genes, a majority of genes were differentially expressed between young and expanded leaves in each species (differential analysis using EBSeq with a PPDE ≥0.95 and a minimal fold change of 2). PT, post-transcriptionally regulated.

The EISA method was used on all expressed genes to identify post-transcriptional events occurring between young and expanded leaves in both C_3_*T. hassleriana* and C_4_*G. gynandra* (Fig. 2A, B). This method requires complete coverage in exons as well as introns. In order to limit false positives, a stringent minimal read cut-off was applied (see Materials and methods for details). This filtering left a quarter of the genes for both species: 4 769 and 5 720 in *G. gynandra* and *T. hassleriana*, respectively(Table 1). Of these genes, 678 in *G. gynandra* and 263 genes in *T. hassleriana* were most likely post-transcriptionally regulated (EISA positive) between young and expanded leaves with a false discovery rate FDR ≤0.05 (Table 1). Finally, 49 post-transcriptionally regulated genes were common to both species, including, for example, the gene encoding phosphoribulokinase (PRK) (Fig. 2C; Supplementary Table S3). This is consistent with a global rewiring of gene expression at the post-transcriptional level specific to the C_4_ leaf. A majority of the predicted post-transcriptionally regulated genes: 349 (51%) for *G. gynandra* and 116 (44%) for *T. hassleriana* were also significantly over-expressed during leaf ontogeny (Fig. 2D). Gene Ontology (GO) terms associated with photosystems and the CBC were significantly over-represented in genes that were post-transcriptionally regulated between young and expanded leaves from *G. gynandra*.

This approach was further corroborated by looking at genes that were previously shown to be under post-transcriptional regulation, for example, maize glutathione reductase 1 (*GR1*) is under post-transcriptional regulation in bundle-sheath cells (Pastori *et al.*, 2000). In *G. gynandra*, *GR1* is under post-transcriptional regulation according to the EISA prediction. A significant drop in ΔExon during leaf development (ΔExon–ΔIntron=–2.66; Supplementary Table S4) was detected. BS-specific post-transcriptional regulation of *GR1* might result from convergent evolution or is ancestral to the monocot/dicot divergence as previously suggested for some C_4_ genes (Aubry *et al.*, 2014*a*).

### *C_4_ cycle genes are mostly transcriptionally regulated during* G. gynandra *leaf ontogeny*

**Fig. 3.**
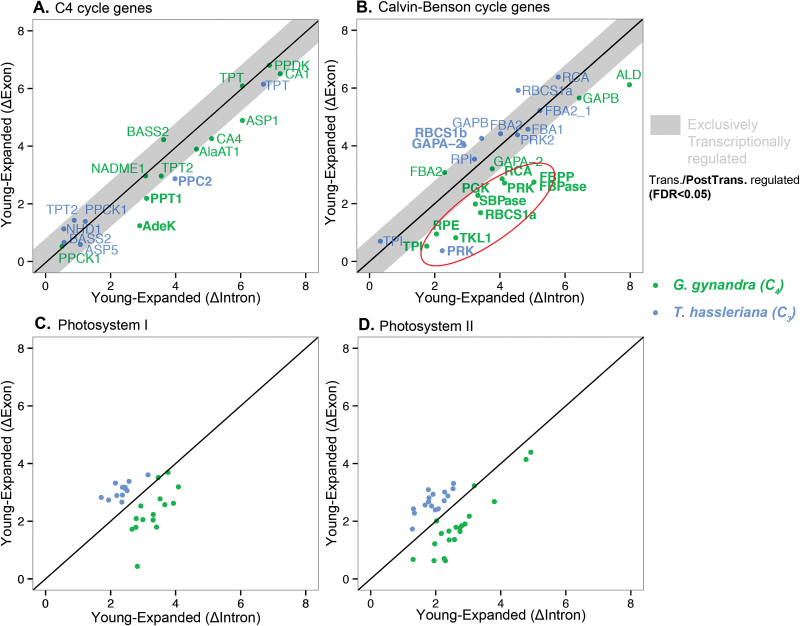
Changes in intronic and exonic levels during leaf development for genes involved in specific pathways. Genes encoding for the C_4_ cycle (A), the Calvin–Benson cycle (B), photosystem I (C), and photosystem II (D) show various levels of post-transcriptional regulation between young and expanded leaves in *G. gynandra* (green dots) and *T. hassleriana* (blue dots). The grey area contains genes that are transcriptionally regulated, i.e. with ΔExon and ΔIntron correlated during leaf development. The names of post-transcriptionally regulated genes are in bold and EISA-positive genes from the Calvin–Benson cycle are highlighted within a red circle. The dots in plot (C) represent subunits of photosystem I: *Lhca1, Lhca2, Lhca3, Lhca4, Lhca5, Lhca6, Ohp2, PsaN, PsaL, PsaD1, Ptac8, PsaE2, PsaG, PsaH2, PsAf, PsaK, PsaO, PsaD2*; and those in plot (D) represent subunits of photosystem II: *Lhcb6, Cab1, Lhcb2.1, Lhcb4.3, Lhcb5, Lhcb4.1, Lhcb3, Psb27, Lpa19, Psbp-1, Pql1, Npq4, PsbY, PsbR, Pql3, PsbX, PsbW, Ppl2, PsbO2, PsbQ, Psb28, Ohp, Lpa2* (for single gene ΔExon and ΔIntron data, see Supplementary Table S4). Abbreviations: AdeK, adenylate kinase; AlaAT1, alanine aminotransferase; ALD, aldolase; ASP1/5, aspartate aminotransferase 1/5; BASS2, bile acid symporter; CA1/4, carbonic anhydrase 1/4; FBA2_1, fructose *bis*phosphate aldolase 2_1; FBPase, fructose-*bis*phosphate aldolase; FBPP, fructose 1,6-*bis*phosphate phosphatase; GAP-A -B, GADPDH subunit-A or –B; NADME1, NAD-dependent malic enzyme 1; NHD1, sodium:proton antiporter; PGK, phosphoglycerate kinase; PPC2, phospho*enol*pyruvate carboxylase; PPCK1, phospho*enol*pyruvate carboxykinase 1; PPDK, pyruvate-orthophosphate dikinase; PPT1, phosphoenolpyruvate/phosphate translocator; PRK1/2, phosphoribulokinase; SBPase, sedoheptulose-*bis*phosphatase; RBCS1a/b, RuBisCO small subunit 1a/b; RCA, RuBisCO activase; RPE, d-ribulose-5-phosphate-3-epimerase; RPI, ribose-5-phosphate isomerase; RuBisCO, ribulose 1,5-*bis*phosphatase carboxylase; TKL1, transketolase 1; TPI, triose phosphate isomerase; TPT1 & 2, triose phosphate/phosphate translocator.

Expression of genes encoding proteins of the C_4_ cycle is up-regulated during leaf development in both species, but to a much larger extent in the C_4_ species (see Supplementary Fig. S1A at *JXB* online). Increased expression of C_4_ cycle genes in the C_3_ species during leaf development has previously been shown in many other dicotyledonous systems. This suggests that C_4_ gene regulatory networks pre-existed in C_3_ species and were associated with their ancestral anaplerotic function (Aubry *et al.*, 2011; Wang *et al.*, 2013). The EISA model tries to identify genes that show expression changes at the exon level that are not accounted for at the intron level. Genes encoding C_4_ core-cycle proteins are not post-transcriptionally regulated: variations of the number of reads that map introns and exons are correlated for most of these genes except for two, *GgPPT1* (phosphoenolpyruvate/phosphate translocator) and *ThPPC2* (phosphoenolpyruvate carboxylase 2) (Fig. 3A). This shows that, in both C_3_ and C_4_ species, C_4_ cycle genes are subjected to an increase in amplitude as the leaf matures but the magnitude of this amplitude is much higher in C_4_ leaves.

Interestingly, this increase in expression does not seem to be regulated at the post-transcriptional level according to EISA. This is consistent with data previously published suggesting a strong involvement of the transcriptional machinery to the C_4_ cycle (Bräutigam *et al.*, 2010; Aubry *et al.*, 2014*a*). However, regulation of M specificity of C_4_ cycle genes like *GgPPDK* (pyruvate-orthophosphate dikinase) or *GgCA4* (β-carbonic anhydrase 4) is regulated by post-transcriptional mechanisms involving untranslated regions (Williams *et al.*, 2016). This suggests a complex interplay between transcriptional and post-transcriptional regulation of gene expression for these genes.

### Calvin–Benson cycle and photosystem genes are post-transcriptionally regulated in the C_4_ leaf gradient

Genes encoding proteins involved in the CBC are up-regulated during leaf development in both C_3_ and C_4_ species (Supplementary Fig. S1). In contrast to the core C_4_ cycle, there is a tendency towards a lower expression of CBC genes in C_4_ leaves (Bräutigam *et al.*, 2010). In *G. gynandra*, most genes (10 out of 13 detected by EISA) showed a significant (FDR ≤0.05) drop in ΔExon compared with ΔIntron (Fig. 3B). In most C_4_ species, the CBC is split between BS and M cells (Majeran *et al.*, 2005, 2010; Aubry *et al.*, 2014*a*; John *et al.*, 2014). Interestingly, all genes involved in the CBC known to be specifically expressed in BS cells are predicted as being post-transcriptionally regulated (FDR ≤0.05, Fig. 3B). Genes that are preferentially expressed in M, like triose phosphate isomerase (*TPI*) or the GAPDH subunit B (*GAP-B*) are also post-transcriptionally regulated, suggesting this mechanism is not exclusive to BS genes. These genes, nonetheless, have their expression level increased at the tissue level, proportional to the leaf age (Supplementary Fig. S1). We postulate that the significant decrease in ΔExon during leaf development is a mixture of transcriptional and post-transcriptional regulation. None of the *T. hassleriana* CBC genes, except phosphoribulokinase (*PRK*), were predicted to be under post-transcriptional regulation during leaf development (blue dots in Fig. 3B). This suggests a post-transcriptional regulation mechanism specific to the C_4_ leaf. It is known that most CBC genes are expressed cell-specifically in *G. gynandra* leaves. Therefore, we propose a model of post-transcriptional regulation that operates between BS and M cells on a specific subset of transcripts. For example, sedoheptulose-*bis*phosphatase (*SBPA*) is mostly expressed in BS cells and the ΔExon/Δintron correlation is lost during leaf development (Fig. 3B). This can be interpreted as an increase in expression at the transcriptional level in BS cells, combined with a mesophyll-specific degradation of exon-only mature transcripts. In a similar manner, a large proportion, i.e. 18 and 23 transcripts encoding for PSI and PSII proteins, respectively, were post-transcriptionally regulated (decrease in ΔExon, Fig. 3C, D). These results indicate that regulation of C_4_ core genes is somehow distinct from photosynthetic genes during C_4_ leaf ontogeny and that this mechanism could be the basis for the actual cell-specific regulation of photosynthesis in C_4_ leaves.

### Post-transcriptional regulation of RbcS and cell-specificity

RuBisCO is a hexadecameric protein, composed of eight nuclear-encoded subunits (RBCS) and eight chloroplast-encoded subunits (RBCL). In C_4_ dicots like *Flaveria* and amaranths, *RbcL* and *RbcS* expression are tightly regulated and repressed in the mesophyll (Patel and Berry, 2008; Berry *et al.*, 2016). *RbcS* expression at the translational level appears to be central to the early stage of development, whereas mRNA stability mediates cell-specificity at a later stage (Patel and Berry, 2008). Consistent with this, ectopic expression of *RbcS* under a constitutive promoter in maize failed to accumulate transcripts in the mesophyll compartment (Wostrikoff *et al.*, 2012).

Our data imply that, despite an overall increase in *RbcS* expression on the whole leaf scale (Supplementary Fig. S1), at least part of the *RbcS1a* expression is under post-transcriptional regulation in *G. gynandra* (Fig. 3B). Given the high specificity of *RbcS* transcripts in the BS compartment at a steady-state level in *G. gynandra* (Aubry *et al.*, 2014*a*), the mesophyllic compartment is the most obvious place for this to happen. The cytosolic degradation of mature transcripts translates to a decrease in ΔExon during leaf development. The potential influence of cytosol-based regulation of *RbcS* transcript levels on chloroplast expression of *RbcL* remains to be shown, as gene expression of both subunits is tightly linked (Wostrikoff *et al.*, 2012).

### Models for post-transcriptional regulation of photosynthesis in C_4_ leaves

We have shown here that post-transcriptional control of RNA stability is likely to play a key function during C_4_ leaf ontogeny, but the molecular basis of the mechanism involved in C_4_ species remains unclear. Our results confirm a large body of data indicating that post-transcriptional regulation is crucial for photosynthesis regulation as, for example, in the case of ferredoxin-1 mRNA stability controlled by light in tobacco (Petracek *et al.*, 1998). In plants, multiple levels of control can regulate cytosolic mRNA stability: basal RNA decay machineries, sequence- and secondary structure-specific decay (that often implies *cis*-elements in untranslated regions), and stimulus-dependent degradation often linked to translational repression (Gutiérrez *et al.*, 1999). The mRNA decay processes are complex and can be subjected to multiple modes of degradation: de-adenylation of the poly-A tail and/or 5′-end decapping and 3′-end uridylation potentially followed by 5′–3′ decay or 3′–5′ degradation by the exosome (Gutiérrez *et al.*, 1999; Siwaszek *et al.*, 2014). All of these mechanisms require a specific array of *trans*-acting factors. We discuss here the likelihood of each potential control mechanism to be involved in the regulation of C_4_ leaf ontogeny.

#### miRNA

miRNA-mediated degradation that is known to repress gene expression in diverse developmental processes (phase transition, leaf shape, floral organ identity; Chen, 2009) could be a possible mechanism to explain the observed post-transcriptional regulation. Unfortunately, no cell-specific miRNA profiling of C_4_ leaves has been published to our knowledge.

#### *Non-sense-mediated decay (NMD*)

Cytosolic aberrant mRNAs may be degraded via the non-sense-mediated mRNA decay (NMD) pathways, considered to be one of the main post-transcriptional regulation processes in eukaryotes (Siwaszek *et al.*, 2014; Dai *et al.*, 2016). However, genes involved in the NMD pathway in plants such as up-frameshift protein 1 and 3 (*UPF1* and *3*) and exoribonucleases (*XRN4, 5,6*) were not differentially expressed between BS and M cells in mature *G. gynandra* leaves and no cell-specific polymorphism could be observed between the transcripts from the two cell types (Aubry *et al.*, 2014*a*). This mechanism is therefore unlikely to be recruited for transcriptional regulation of photosynthetic genes during C_4_ leaf ontogeny.

#### Translation

Interaction with the translational machinery is another way of controlling mRNA levels by translational repression (Roy and Jacobson, 2013). The relationship between mRNA decay and translation efficiency are, however, complex and differ on a gene-to-gene basis. More work is necessary to show whether ribosomal loading of mRNA is actually different in genes predicted to be post-transcriptionally regulated using EISA modelling. Interestingly, a complex regulation of translation of *RbcS* transcripts observed in Arabidopsis (assessed by monitoring the association of transcripts to various polysomal fractions) may indicate that already in C_3_ ancestors *RuBisCO* expression is under a specific regime of transcriptional and post-transcriptional regulation (Piques *et al.*, 2009). Therefore, further experiments analysing the association of photosynthesis genes with ribosomes on a cell-specific basis are required. Moreover, applying recently developed ribosomal footprints to such leaf material (Ingolia *et al.*, 2009; Juntawong *et al.*, 2014) could shine new light on transcription–translation interactions in a C_4_ context.

#### Splicing

Differences in mRNA splicing at the pathway level that would lead to a mis-interpretation of the variations in intronic signals are unlikely. Indeed, a large proportion of genes predicted to be post-transcriptionally regulated and involved in the same pathway also present very distinct intron/exon organization (Gaidatzis *et al.*, 2015). The median number of exons and introns of post-transcriptionally (EISA FDR ≤0.05) or transcriptionally regulated (EISA FDR >0.05) genes were compared in each species and showed no significant differences based on Wilcoxon–ann–Whitney test. This indicates that the EISA output is not biased by the structures of the genes.

#### Post-transcriptional co-ordination of gene expression

In *G. gynandra*, M specificity of two C_4_ genes, *PPDK* and *CA4*, is mediated by 5′ and 3′ UTR regions (Kajala *et al.*, 2012; Williams *et al.*, 2012, 2016). The mechanism co-ordinating the cell-specific expression involves a *cis*-element (MEM2) that specifically increases translation in the M compartment (Williams *et al.*, 2016). Other examples of gene expression co-ordination of functionally related genes have been described (Keene, 2007; Goodarzi *et al.*, 2012). Here we postulate that mechanisms similar to those co-ordinating the expression of photosynthesis genes have been recruited during C_4_ leaf development. More work is necessary to identify which of the 200 predicted RNA-binding proteins from six different families in Arabidopsis might regulate mRNA stability (Fedoroff, 2002; Ambrosone *et al.*, 2012).

Finally, a model involving RNA binding proteins that regulate the mRNA stability of a given set of photosynthesis-related genes during leaf ontogeny of C_4_ might be the simplest scenario that explains our observations (Williams *et al.*, 2012). Distinct families of RNA binding proteins could be involved (Silverman *et al.*, 2013), such as PUF proteins that often recognize 3′ UTRs and are dependent on secondary RNA structure (Francischini and Quaggio, 2009; Lu *et al.*, 2009) or sequence-specific PENTATRICOPEPTIDE REPEAT (PPR) that are mostly but not exclusively localized in mitochondria and chloroplasts (Colcombet *et al.*, 2013). More work is required to understand the extent of its complexity (e.g. timing of the regulation in both cells) and diversity (e.g. how that regulation accommodates multiple types of Kranz anatomy) across other C_4_ species. Interestingly, this model would provide two advantages at the pathway level, (i) a reasonable control over transcripts originating from M cells in M cells themselves, and, (ii) would avoid the potential accumulation of transcripts that could have leaked from BS cells (or other cells) into M cells and is, therefore an efficient way to control translation tightly in each respective compartment.

### EISA limitations and potentials for the C_4_ photosynthesis field

Evidence of the presence of unspliced pre-mature RNAs in total RNA samples has already been reported when comparing transcriptome and polyribosomal translatome fractions from Arabidopsis leaves (Aubry *et al.*, 2014*b*; Zhang *et al.*, 2015). When the fraction of RNA that binds ribosomes (referred to as the ‘translatome’) was immunopurified, most of the intron-containing pre-mRNAs were eliminated. This also implies that the EISA method cannot be applied to data deriving from TRAPseq experiments recently used in one of the few cell-specific expression-profiling studies of C_3_ BS cells from Arabidopsis (Aubry *et al.*, 2014*b*).

Using data from entire leaf tissue might not be sufficient to obtain a precise cell-specific understanding of the post-transcriptional mechanisms, but does provide a genome-wide insight of their involvement in the pathway regulation. Further experiments using cell-specific fractions with deep sequencing coverage, and taking into account the untranslated regions of the genes for the EISA, are needed to refine the EISA analysis.

## Conclusions

Relatively few genes are known to be post-transcriptionally regulated in C_4_ leaves and the extent to which this level of regulation is involved in cellular fate remains unclear. Some level of post-transcriptional regulation has been suggested to occur in a single gene-based manner, such as for *CAs*, *PPDK*, and *RbcS* that are specific to C_4_ leaves of multiple genes encoding photosynthetic machinery components. The model proposed here is reminiscent of the theory that conceptualizes high-order regulation of functionally related mRNAs (Keene, 2007; Tenenbaum *et al.*, 2011). More work is needed to clarify the exact significance of this post-transcriptional signature in each cell type, in particular, the mesophyll compartment of C_4_ leaves and how this mechanism interacts with predicted transcriptional modules and translation (Williams *et al.*, 2012; Aubry *et al.*, 2014*a*; Külahoglu *et al.*, 2014). Perhaps not surprisingly, the stability of chloroplast-encoded transcripts is also very variable among the developmental stages (Klaff and Gruissem, 1991). The fact that a large majority of the nuclear-encoded proteins in the C_4_ leaf under post-transcriptional regulation are eventually targeted to the chloroplast might suggest an involvement of some plastid-to-nucleus retrograde signalling in this process. Unfortunately, all high-throughput C_4_ cell-specific data available (Li *et al.*, 2010; Chang *et al.*, 2012; Aubry *et al.*, 2014*a*; John *et al.*, 2014) do not have enough coverage to use the EISA method robustly (data not shown). Therefore, in the context of C_4_ engineering, more research is required to identify relevant RNA binding, RNA processing (helicases) proteins, and non-coding regulatory RNA. Together with a careful monitoring of mRNA half-lives, this could set the stage for the engineering of an efficient C_4_ cycle in a C_3_ species such as rice. Our unbiased genome-wide approach shows a massive rewiring of C_4_ leaves at the post-transcriptional level compared with C_3_ leaves. It may be crucial to understand this mechanism before trying to transform C_3_ into C_4_ crops.

## Supplementary data

Supplementary data can be found at *JXB* online.

Figure S1. Gene expression profiles in leaf gradient of *T. hassleriana* and *G. gynandra* for (A) C_4_ cycle genes and (B) the Calvin–Benson cycle.

Table S1. EISA analysis on a control dataset from the WT and an *ago1-27* Arabidopsis mutant.

Table S2. List of transcripts that are differentially expressed between young and expanded leaves in *G. gynandra* and *T. hassleriana*. 

Table S3. List of transcripts that are common to both species after EISA analysis during leaf development (described by their AGI reference number).

Table S4. List of transcripts that are predicted to be post-transcriptionally regulated by EISA between young and expanded leaves in *G. gynandra* and *T. hassleriana*.

## Supplementary Material

Supplementary_Figure_S1

Supplementary_Table_S1

Supplementary_Table_S2

Supplementary_Table_S3

Supplementary_Table_S4
