# Supplementary material for: Post-transcriptional regulation of photosynthetic genes is a key driver of C4 leaf ontogeny
Source: J Exp Bot. 2016 Oct 18;68(2):137–46. doi: 10.1093/jxb/erw386 (PMC5853474; doi:10.1093/jxb/erw386)
Supplement: Supplementary_Figure_S1 [file erw386_suppl_supplementary_figure_s1.pdf]

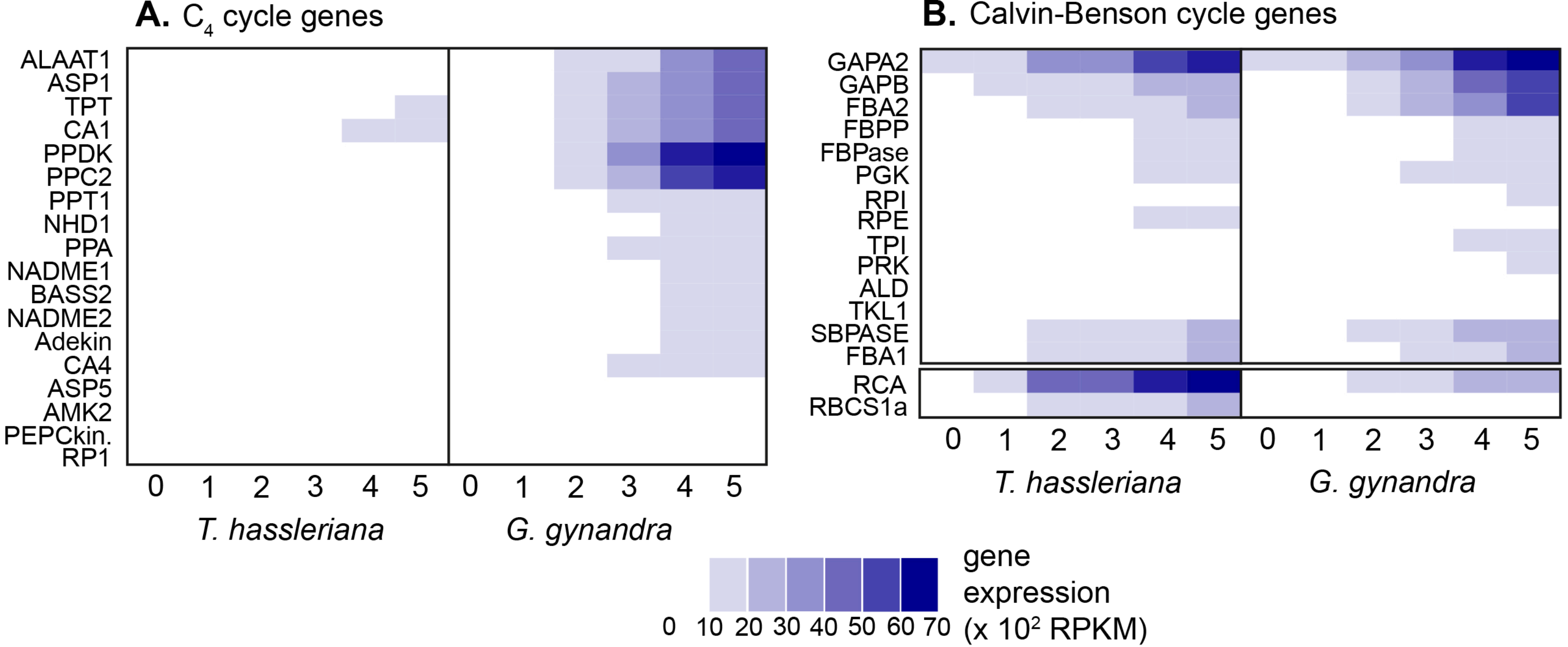

Supplementary Fig. 1. Gene expression profiles in leaf gradient of *T. hassleriana* and *G. gynandra* for (A) C_4 cycle genes and (B) the Calvin Benson cycle. Expression values were extracted from Külahoglu et al. (Külahoglu et al., 2014) and expressed in reads per kilobase per million mapped reads (RPKM). Stages 0 to 5 represent leaf stage at different age, from the younger to the older. For the EISA analysis, only the extreme stages 0 and 5 of the gradient were taken into consideration.
